# Impact of Quorum Sensing Molecules on Plant Growth and Immune System

**DOI:** 10.3389/fmicb.2020.01545

**Published:** 2020-07-16

**Authors:** Abhishek Shrestha, Maja Grimm, Ichie Ojiro, Johannes Krumwiede, Adam Schikora

**Affiliations:** ^1^Institute for Epidemiology and Pathogen Diagnostics, Federal Research Centre for Cultivated Plants, Julius Kühn-Institut, Braunschweig, Germany; ^2^Faculty of Agriculture, Shizuoka University, Shizuoka, Japan

**Keywords:** AHL, quorum sensing, priming, growth promotion, resistance induction

## Abstract

Bacterial quorum-sensing (QS) molecules are one of the primary means allowing communication between bacterial cells or populations. Plants also evolved to perceive and respond to those molecules. *N*-acyl homoserine lactones (AHL) are QS molecules, of which impact has been extensively studied in different plants. Most studies, however, assessed the interactions in a bilateral manner, a nature of interactions, which occurs rarely, if at all, in nature. Here, we investigated how *Arabidopsis thaliana* responds to the presence of different single AHL molecules and their combinations. We assumed that this reflects the situation in the rhizosphere more accurately than the presence of a single AHL molecule. In order to assess those effects, we monitored the plant growth and defense responses as well as resistance to the plant pathogen *Pseudomonas syringae* pathovar *tomato* (*Pst*). Our results indicate that the complex interactions between multiple AHL and plants may have surprisingly similar outcomes. Individually, some of the AHL molecules positively influenced plant growth, while others induced the already known AHL-priming for induced resistance. Their combinations had a relatively low impact on the growth but seemed to induce resistance mechanisms. Very striking was the fact that all triple, the quadruple as well as the double combination(s) with long-chained AHL molecules increased the resistance to *Pst*. These findings indicate that induced resistance against plant pathogens could be one of the major outcomes of an AHL perception. Taken together, we present here the first study on how plants respond to the complexity of bacterial quorum sensing.

## Introduction

Interactions between plant and the associated bacteria are based on an exchange and perception of diverse molecules that are both plant- and bacteria-originated. In the last decade, bacterial quorum sensing molecules were shown to play a crucial role in the communication between the associated bacterial community and the host plant.

Quorum sensing (QS) was discovered as a means of communication within bacterial populations; it is a process based on the synthesis and detection of autoinducer or QS molecules. This phenomenon enables bacteria to monitor the cell density and to coordinate collective changes in behavior. Gram-negative bacteria generally rely on the synthesis of autoinducers like *N*-acyl homoserine lactones (AHL) or cyclodipeptides. Perception of QS molecules in bacteria activates or deactivates transcription of numerous QS-regulated genes including virulence factors, biofilm formation, chemotaxis, and many more (Bellezza et al., 2014). AHL is one of the major and most extensively studied class of QS molecules. Molecules from this group are comprised of two moieties: a homoserine lactone ring and an acyl side-chain, ranging from 4 to 18 carbons. The acyl chain may vary in the length or in the substitution of the hydrogen at the C-3 position with a hydroxyl or a ketone group (Whitehead et al., 2001; Marketon et al., 2002; Von Bodman et al., 2003). The precise recognition of the AHL by its cognate receptor depends on the lactone ring; the amide group and the fatty acid chain length that together determine the specificity of the cell-to-cell recognition and interaction (Churchill and Chen, 2011).

Since bacteria very often interact with other organisms, it is maybe not surprising that QS molecules may modulate the behavior of other bacterial species and even higher organisms (Williams, 2007). Even though it is still not clearly understood how plants perceive these signaling molecules, the impact of AHL was reported on many occasions including changes in the expression of various genes, proteomes and root development (Mathesius et al., 2003; Ortiz-Castro et al., 2008; Von Rad et al., 2008; Schikora et al., 2011; Schenk et al., 2012). The first evidence of the impact of bacterial AHL on plants was presented by Mathesius et al. (2003). In this study, proteomic analysis showed significant differences in the abundance of more than 150 proteins involved mainly in physiological activities of *Medicago truncatula* including flavonoid synthesis, hormone metabolism and oxidative stress in the roots when treated with two different AHL (oxo-C12-HSL and oxo-C16-HSL). Similarly, in response to the AHL, oxo-C8-HSL, *Arabidopsis* seedlings showed differences in the accumulation of proteins that were involved in carbon metabolism, protein biosynthesis and plant resistance (Miao et al., 2012; Ding et al., 2016).

Multiple studies suggested that plant responses to a particular AHL molecule are very specific and depend on the length of the acyl moiety. AHL with a short acyl chain length of 4 to 6 carbons have been shown to increase primary root elongation and growth rate. These effects were mainly attributed to changes in auxin level (Von Rad et al., 2008; Bai et al., 2012; Liu et al., 2012; Schenk et al., 2012; Zhao et al., 2016). Treatment with oxo-C6-HSL resulted in the expression of genes predominantly associated with auxin and cytokinin signaling pathways (Von Rad et al., 2008; Zhao et al., 2016). Auxin was also shown to be involved in the formation of AHL-induced adventitious roots in *Vigna radiata* (Bai et al., 2012). In a similar study, treatment with oxo-C10-HSL induced formation of adventitious roots in mung bean via an H_2_O_2_- and NO-dependent cyclic GMP-signaling (Bai et al., 2012). Nonetheless, the formation of lateral roots in *Arabidopsis* due to pretreatment of oxo-C10-HSL seems independent of auxin concentration (Ortiz-Castro et al., 2008). Palmer et al. (2014) argued that AHL-dependent growth induction is associated with alteration in transpiration intensity.

Apart from the AHL-induced plant growth and changes in root architecture, AHL may elicit changes in defense mechanisms. AHL with a long acyl chain length of 12 to 16 carbon atoms were shown to induce AHL-priming for enhanced resistance in *M. truncatula, Arabidopsis thaliana*, and *Hordeum vulgare* (Schikora et al., 2011; Schenk et al., 2012, 2014; Zarkani et al., 2013; Shrestha et al., 2019). The systemic effect of enhanced resistance in AHL-primed plants is notably associated with salicylic acid signaling. In addition, AHL-priming with oxo-C14-HSL induced oxylipin accumulation in distal tissues of *Arabidopsis* that promoted stomatal closure and accumulation of callose and phenolic compounds like e.g., lignin in cell walls, resulting in enhanced resistance toward bacterial and fungal pathogens (Schenk and Schikora, 2014). Very interesting is the fact that different plants may respond differently to the same AHL molecule. For example, oxo-C14-HSL synthesized by *Ensifer meliloti* stimulated root nodulation in *M*. *truncatula* (Veliz-Vallejos et al., 2014) whereas in *Arabidopsis* and barley, it enhanced resistance against *Pseudomonas syringae* and *Blumeria graminis*, respectively (Schikora et al., 2011; Schenk et al., 2012, 2014; Zarkani et al., 2013; Shrestha et al., 2019).

Despite the numerous reports on the linear interactions between specific AHL molecules and the plant, studies on interaction between the host plant and complex AHL combination are missing. Therefore, in this study we investigated how plant responds to complex AHL combinations, in which not a single but multiple AHL molecules are present. We assumed that this reflects the situation in the rhizosphere more accurately than the presence of a single AHL molecule. We monitored the plant growth and defense responses as well as resistance to the plant pathogen *P. syringae* pathovar *tomato* (*Pst*). Our results indicate that the complex interactions may have surprisingly similar outcomes. Whereas the presence of single AHL molecules induces changes in the growth and AHL-priming, known already from previous reports, the combinations had relatively low impact on the growth but seemed to induce resistance mechanisms. We present here the first data on how a plant respond to the complexity of bacterial quorum sensing.

## Materials and Methods

### Plant Growth

Wild-type *A. thaliana Col-*0 seeds were surface sterilized by washing with sterilization mix of 12% bleach: deionized water: 100% EtOH (1:3:4) for 10 min and further rinsed twice with 100% EtOH for 1 min. The seeds were dried and placed on 1/2-strength MS (Murashige and Skoog) agar plates under sterile conditions. For *P. syringae* pathogenicity assay, root growth and biomass assays, 1-week old seedlings were transferred to square Petri dishes containing 50 ml of 1/2-strength MS medium with different *N*-acyl homoserine lactones (AHL) (Sigma-Aldrich) or their possible combinations, or acetone as a control, and were allowed to grow vertically (see below). Plants were grown at controlled conditions: day/night 8/16 h photoperiod and 22°C, light intensity of 150 μmol/m^2^s and 60% humidity, in a growth chamber for 3 weeks. For gene expression analysis, 2-week old seedlings were transferred to six-well plates with 3 ml 1/2-strength MS medium per well 1 day prior to pretreatment with AHL.

### Pretreatment With AHL

*Arabidopsis Col*-0 seedlings grown on 1/2-strength MS medium under sterile conditions were transferred to square Petri dishes or six-well plates containing 1/2-strength MS medium with different AHL (Schikora et al., 2011): *N*-(3-oxohexanoyl)-*L*-homoserine lactone (oxo-C6-HSL), *N*-(3-oxooctanoyl)-*L*-homoserine lactone (oxo-C8-HSL), *N*-(3-oxododecanoyl)-*L*-homoserine lactone (oxo-C12-HSL) and *N*-(3-oxotetradecanoyl)-*L*-homoserine lactone (oxo-C14-HSL) (Sigma-Aldrich) and their possible combinations at a final concentration of 6 μM. Oxo-C6-HSL, oxo-C8-HSL, oxo-C12-HSL and oxo-C14-HSL were dissolved prior in acetone to acquire a stock solution of 60 mM. The seedlings on square Petri dishes were grown for 3 weeks whereas the seedlings in six-well plates were grown for additional 3 days.

### Root Elongation and Biomass Assay

In order to reveal the differences in growth parameters of *Arabidopsis* seedlings due to different AHL-treatments, 1-week old seedlings were transferred to Petri dishes containing 1/2-strength MS medium with different AHL, either individually or in combinations or acetone as a control. The seedlings were allowed to grow vertically in controlled conditions for three additional weeks. For the root elongation analysis, the length of each root was measured manually. For biomass analysis, three to five plants from each plate were pooled together and weighed.

### Gene Expression Analysis

In order to induce AHL-priming, *Col*-0 *Arabidopsis* seedlings grown on 1/2-strength MS medium under sterile conditions were transferred to six-well plates with 3 ml 1/2-strength MS medium 24 h prior to pretreatment with AHL at a final concentration of 6 μM. The plants were grown for additional 3 days in the same conditions: day/night 8/16 h photoperiod and 22°C, light intensity of 150 μmol/m^2^s and 60% humidity. All experiments were performed with the solvent control, acetone. To reveal the differences in defense responses between AHL-primed and naïve plants, 3 days after the pretreatment, seedlings were treated with 100 nM flg22. *Arabidopsis* seedlings pretreated with different combinations of AHL were harvested at 0 and 2 h post flg22 treatment. Whole seedlings were homogenized and total RNA was extracted using peqGOLD TriFast (VWR) following manufacturer’s recommendations. RNA concentration and quality were determined using the Nanodrop Bioanalyzer. One μg of total RNA was DNAse digested using the PerfeCTa DNAse I (Quanta Biosciences) and subsequently cDNA synthesis was carried out using the qScript cDNA Synthesis kit (Quanta Biosciences) according to the manufacturer’s recommendations. Quantitative RT-PCR (qPCR) was performed using primers listed in Supplementary Table S1. All expression levels were normalized to the expression of *AtUBQ*. The experiments were performed in a minimum of six independent replications.

### Challenge With *Pseudomonas syringae*

*Pseudomonas syringae* pv. *tomato* DC3000 *(Pst)* was grown on King’s B medium containing selective antibiotics for 2 days at 28°C. The bacteria were washed and resuspended in 10 mM MgCl_2_ and O.D_600_ of the bacterial culture was adjusted to 0.01. Plants growing on different combinations of AHLs were sprayed homogenously with *Pst* at O.D_600_ = 0.01, corresponding to 10^7^ colony forming units (CFU)/ml. Twelve and 96 h post inoculation with *Pst*, three plants were pooled, weighed and then homogenized in 10 mM MgCl_2_, serial dilution was plated in duplicates on King’s B agar plates containing selective antibiotics to assess the CFU number. The experiments were performed in six independent replications in two independent experiments.

### Statistical Analysis

Root growth assays were performed in a minimum of 75 biological replicates from five independent experiments. Biomass assays were performed in at least 20 biological replicates from five independent experiments. Quantitative PCR assays were performed in six biological replicates from three biologically independent experiments. *Pst* pathogenicity assay was performed in six biological replicates from two independent experiments. *p*-values < 0.05 in the Student’s *t*-test were considered as indicative for a significant difference. Graphs were made using Prism 7 (GraphPad Software, La Jolla, CA, United States).

## Results

### Bacterial Quorum Sensing Molecules Affect the Plant Growth

**FIGURE 1:**
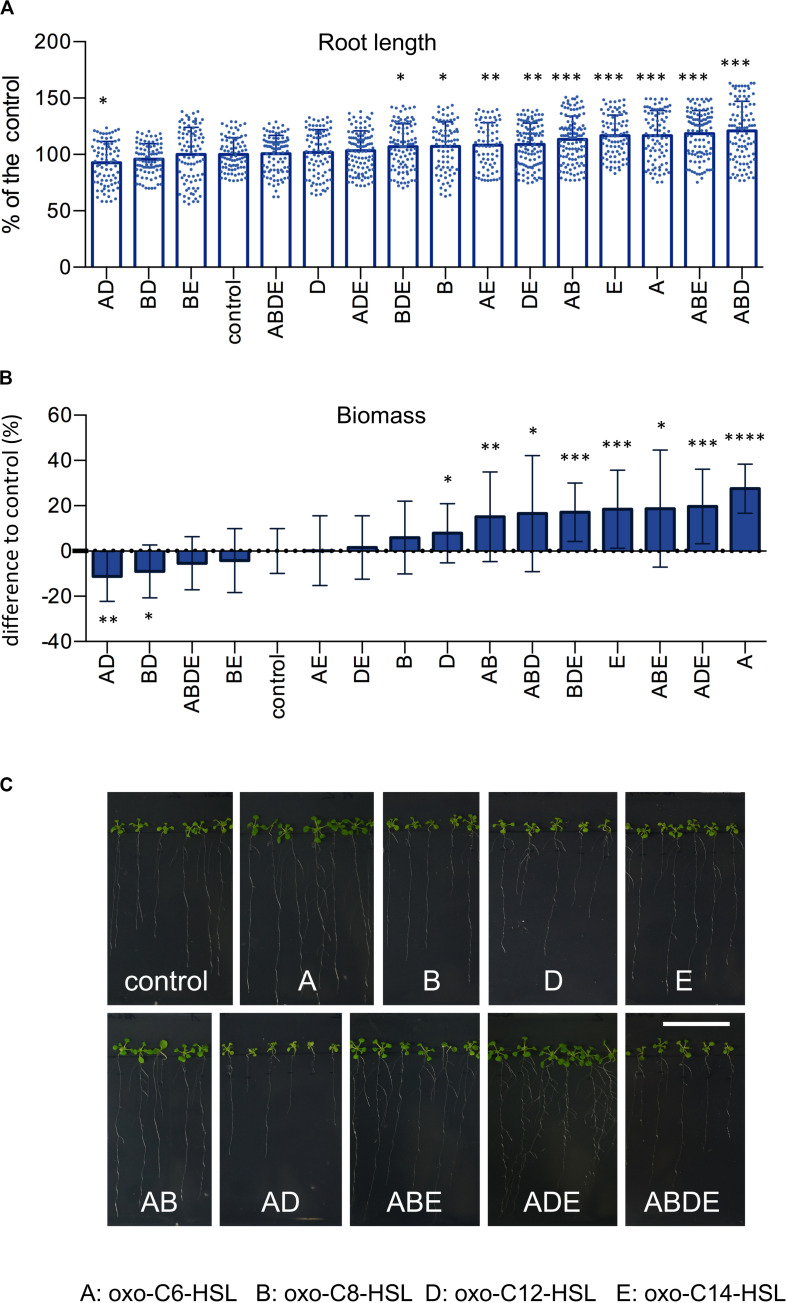
AHL and their combinations have an impact on plant growth. *Arabidopsis thaliana Col*-0 plants were grown under the treatment of 6 μm AHL alone or their combinations for 3 weeks and the root length **(A)** and plant biomass **(B)** were assessed. The control, acetone-treated plants, was set to 100%. * indicates *p* < 0.05, **p < 0.005, ***p < 0.0005, and ****p < 0.00005 in Student’s *t*-test, minim. *n* = 20. Representative photographs presenting the phenotype of *A. thaliana Col*-0 after the exposition to AHL, are presented in **(C)**, the bar represents 3 cm.

In order to assess how the presence of multiple *N*-acyl homoserine lactones (AHL) in the rhizosphere influences the growth of plants, we exposed *A. thaliana Col*-0 seedlings to four AHL molecules (A: oxo-C6-HSL; B: oxo-C8-HSL; D: oxo-C12-HSL and E: oxo-C14-HSL) individually, and in all possible combinations (Supplementary Figure S1A). *Arabidopsis* seeds were germinated on sterile 1/2-strength MS medium for 1 week and transferred to fresh 1/2-strength MS media supplemented with single AHL or their combinations for an additional 3 weeks (Supplementary Figure S1B). Assessment of the root length revealed that while the treatment of plants with combinations AD, BD, BE, ABDE or the single D (oxo-C12-HSL) had no impact on roots length, if compared to the control (Figure 1A). Contrarily, treatments with combinations ABD, ABE, AB or the single A (oxo-C6-HSL) and E (oxo-C14-HSL) molecules, if compared to the control, resulted in longer roots (Figure 1A). The impact of other single or combinations of AHL was less elusive since the treatment resulted in an intermediate root length (Figure 1A).

In addition to root length, we also measured the plant weight. The acquired results revealed that treatment with combinations AD, BD, ABDE, and BE had no impact on plant weight, while treatments with combinations AB, ABD, ABE and single A and E molecules resulted in plant with the highest biomasses (Figure 1B). In addition, we also observed a negative correlation between root length and weight. For instance, treatment with the single molecule D (oxo-C12-HSL) resulted in shorter roots, however, higher plant biomass. The same was true for the treatment with combinations ADE and BDE, which resulted in shorter roots but higher plant biomass (Figure 1B).

Taking together, we observed that some AHL molecules or their combinations were able to significantly enhance root growth or plant biomass, while others, for example combinations AD and BD, negatively influenced plant growth.

### Pretreatment With oxo-C14-HSL Induces AHL-Priming

**FIGURE 2:**
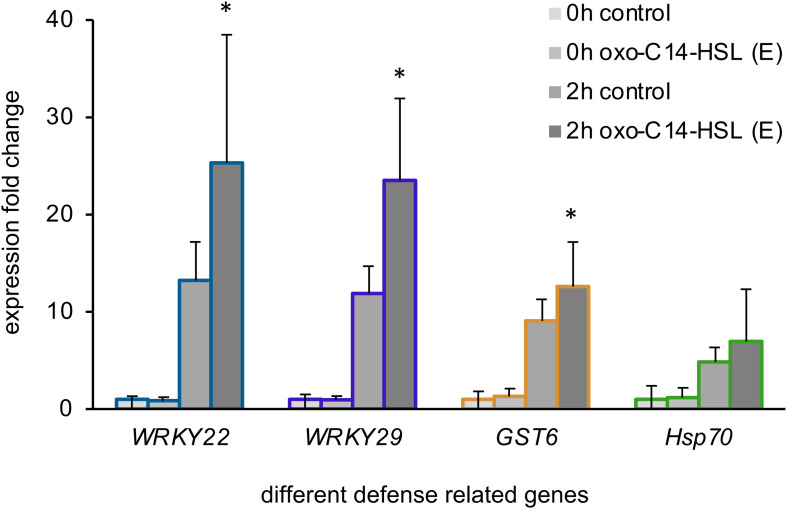
Long-chained AHL, oxo-C14-HSL, induced AHL-priming. Expression levels of four defense-related genes: the transcription factors *WRKY22* and *WRKY29*, the *Glutathione S-Transferase 6 (GST6)* and the *Heat Shock Protein 70 (Hsp70)* assessed in plants pretreated for 3 days with either the acetone solvent (control) or 6 μM oxo-C14-HSL (E), prior (0 h) and 2 h (2 h) after the challenge with 100 nM flg22. The mRNA level in control plants (0 h control) was set to 1, the expression was normalized to the expression levels of housekeeping gene *At5g25760* (Ubiquitin ligase). * indicates *p* < 0.05 in Student’s *t*-test.

In the next step, we wondered if the treatment with the different AHL or their combinations would impact immune responses of *Arabidopsis*. To answer this question, we first monitored the expression of four defense-related genes: *WRKY22*, *WRKY29*, the *Glutathione S-Transferase 6* (*GST6*) and the *Heat Shock Protein 70* (*Hsp70*), in plants pretreated with the AHL solvent control (acetone) or with 6 μM oxo-C14-HSL (E). This AHL is known to induce AHL-priming for enhanced resistance. We assessed the gene expression before (0 h) and 2 h after a challenge with 100 nM flg22 in oxo-C14-HSL pretreated plants (Figure 2). Our results aligned with the findings reported previously for AHL-priming (Schikora et al., 2011; Schenk et al., 2014), we observed higher induction of *WRKY22*, *WRKY29*, and *GST6* expression in AHL-pretreated plants, if compared to the control plants after the challenge with flg22 (Figure 2).

### Pretreatment With AHL Affects the Responsiveness to flg22

**FIGURE 3:**
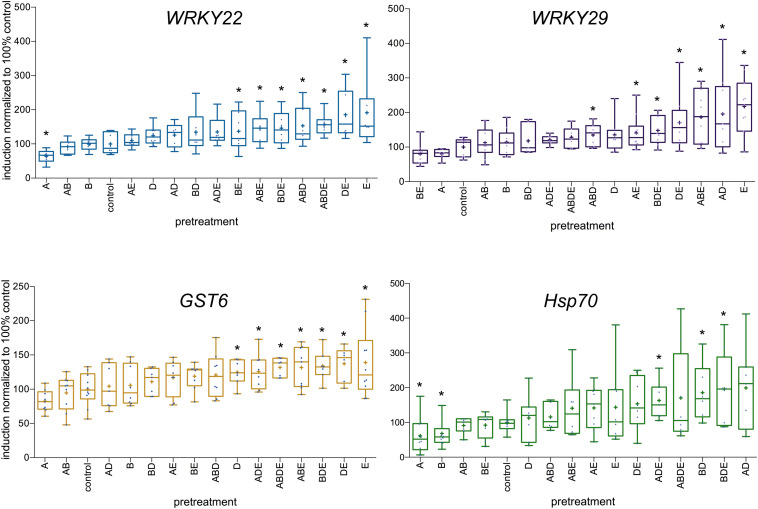
Differences in the induction of defense-related genes upon AHL pretreatment. Single AHL: *N*-(3-oxohexanoyl)-*L*-homoserine lactone (oxo-C6-HSL) (A), *N*-(3-oxooctanoyl)-*L*-homoserine lactone (oxo-C8-HSL) (B), *N*-(3-oxododecanoyl)-*L*-homoserine lactone (oxo-C12-HSL) (D) and *N*-(3-oxotetradecanoyl)- *L*-homoserine lactone (oxo-C14-HSL) (E), as well as their combinations, were used as pretreatment for 3 days prior to the challenge with 100 nM flg22. The induction of the expression of the transcription factors *WRKY22* and *WRKY29*, the *Glutathione-S-Transferase 6 (GST6)* and the *Heat Shock Protein 70 (Hsp70)* was assessed 2 h after the flg22 challenge. The induction in control plants (acetone pretreatment) was set to 100%. The level of expression was normalized to the expression of the housekeeping gene *At5g25760* (Ubiquitin ligase). ^∗^ indicates *p* < 0.05 in Student’s *t*-test.

Following those results, *Arabidopsis* plants were pretreated with all four tested AHL molecules individually as well as in all possible combinations, similar to experiments described above. The expression levels of the four defense-related genes: transcription factors *WRKY22* and *WRKY29*, the *GST6* and *Hsp70*, were assessed 2 h after the challenge with 100 nM flg22. The induction ratio for each gene (difference in gene expression between the 0 h and the 2 h time points) in control plants was set to 100%. The highest induction rates were observed after pretreatment with either the single molecule E (oxo-C14-HSL) or the combinations DE (in case of *WRKY22* and *GST6*) and AD in case of *WRKY29*. On the other hand, the lowest induction rates were observed after pretreatment with the single molecule A (oxo-C6-HSL) in case of *WRKY22* and *Hsp70* and after pretreatment with molecule B (oxo-C8-HSL) in case of *Hsp70* (Figure 3). Very striking in our results was a particular tendency, it appears namely that pretreatment with triple AHL combinations as well as combinations with long-chained AHL (D and E) resulted in higher induction rate of particular genes, than pretreatments with single short-chained AHL (A or B) or their combinations. This was especially apparent for the expression levels of *WRKY22* (Figure 3) and *GST6* (Figure 3).

### Comparison Between Growth and Defense Parameters Reveals That Different Interactions May Result in a Similar Outcome

**FIGURE 4:**
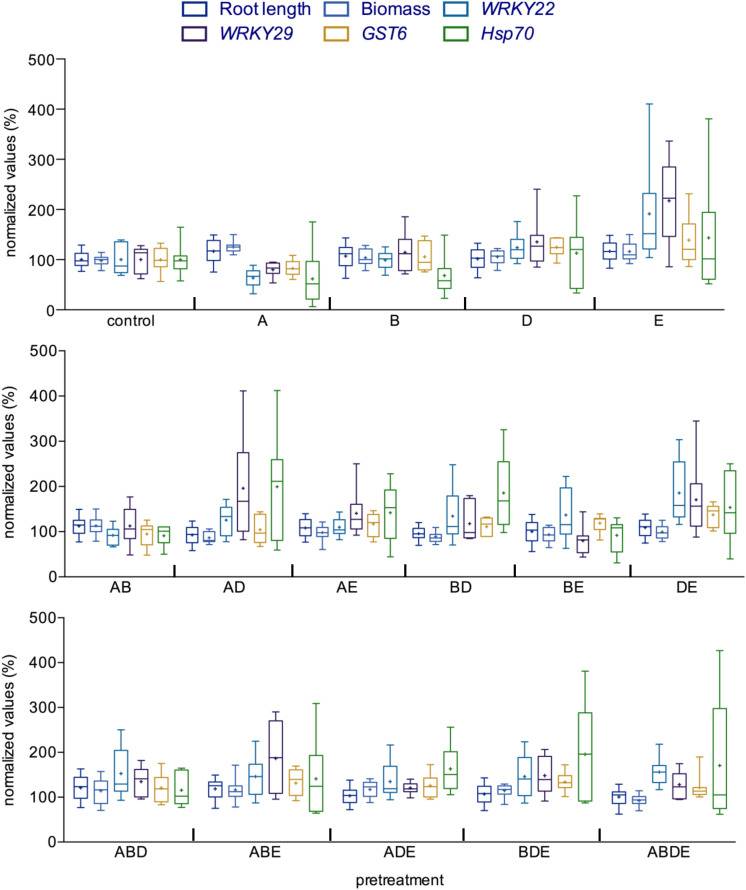
Comparison between growth parameters and immune response indicates significant impact of complex AHL combinations. All parameters measured during this study were compared with each other for each AHL treatment/pretreatment. The values of the control plants were set to 100%. Root length and biomass are presented as a direct ratio of the control values; the gene expression analysis is presented as a percentage of the induction in the gene expression 2 h after challenge with flg22. *WRKY22* and *WRKY29* encode for transcription factors, the *GST6* for the *Glutathione S-Transferase 6* and *Hsp70* for *Heat Shock Protein 70*. A: *N*-(3-oxohexanoyl)-*L*-homoserine lactone (oxo-C6-HSL); B: *N*-(3-oxooctanoyl)-*L*-homoserine lactone (oxo-C8-HSL); D: *N*-(3-oxododecanoyl)-*L*-homoserine lactone (oxo-C12-HSL); and E: *N*-(3-oxotetradecanoyl)-*L*-homoserine lactone (oxo-C14-HSL). Multiple letters indicate the used combination.

The seemingly diverse impact of the pretreatment with AHL molecules or their combinations prompted us to compare the outcome of such interactions in more detail. To this end, we set the root length, biomass as well as the induction of gene expression in the control plants (pretreatment with acetone) to 100% and calculated the results accordingly. Several patterns emerged from such comparison, apparently, pretreatment with the short-chained AHL, oxo-C6-HSL (A) induced the root length and biomass, while pretreatment with the long-chained AHL oxo-C14-HSL (E) enhanced expression of several defense-related genes, as observed after an additional challenge with flg22 (Figure 4; Schenk et al., 2012). In addition, except for the combination of the shortest AHL (AB), all other double combinations seem to enhance the induction of gene expression after flg22 treatment, while having no or only very low impact on the plant growth. Similarly, pretreatments with all triple and the quadruple combinations resulted in AHL-priming for enhanced gene induction as indicated by a higher induction of the expression of the four tested genes, when compared to the induction rate of the control pretreatment after flg22 challenge (Figure 4).

Taken together, the direct comparison indicated that although in bilateral interactions, it is the type of the AHL molecule that decides on the outcome of the interaction, in complex interactions, it is rather the number of different AHL molecules. Pretreatment with multiple AHL molecules induced AHL-priming for enhanced resistance, the only exception was the pretreatment with the combination of short-chained AHL (AB), in this case the AHL-priming was not observed.

### Enhanced Resistance Seems to Be a General Response to Complex AHL Combinations, With Some Exceptions

**FIGURE 5:**
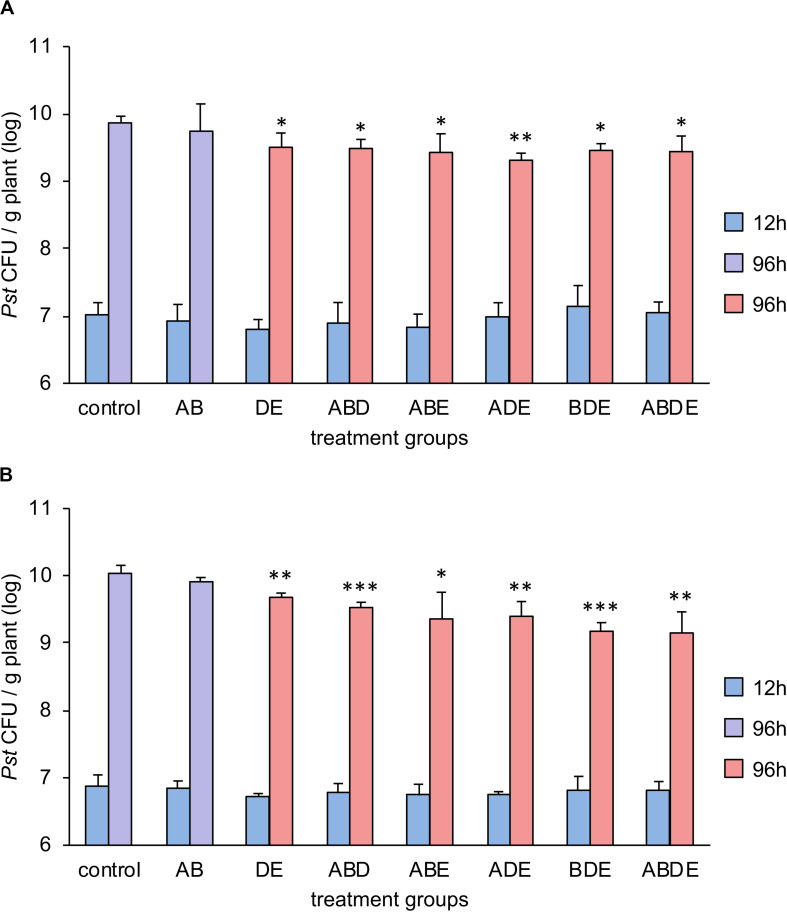
Multiple AHL combinations induce AHL-priming for enhanced resistance against *Pst*. *Arabidopsis thaliana Col*-0 plants were transferred to 1/2-strength MS medium supplemented with combinations of AHL as indicated, 1 week after germination. After additional 3 weeks, plants were challenged with *Pseudomonas syringae* pathovar *tomato (Pst)*. Bacteria were grown on King’s B medium, washed in 10 mM MgCl_2_ prior use and the optical density was adjusted to 0.01 (10^7^ CFU/ml). Bacterial colony forming unit (CFU) number was assessed 12 h and 96 h after the spray-inoculation. A: *N*-(3-oxohexanoyl)-*L*-homoserine lactone (oxo-C6-HSL); B: *N*-(3-oxooctanoyl)-*L*-homoserine lactone (oxo-C8-HSL); D: *N*-(3-oxododecanoyl)-*L*-homoserine lactone (oxo-C12-HSL); and E: *N*-(3-oxotetradecanoyl)-*L*-homoserine lactone (oxo-C14-HSL). Multiple letters indicate the combination used for the pretreatment. Different colors represent the significance groups at *p* < 0.05 in Student’s *t*-test. In addition, ^∗^ indicates *p* < 0.05, ^∗∗^*p* < 0.005 and ^∗∗∗^*p* < 0.0005 in Student’s *t*-test. **(A,B)** Display two independent experiments.

In order to test our hypothesis that the impact of AHL molecules on plant immunity is linked to their complexity rather than specific molecules, we pretreated *Arabidopsis* plants with two double combinations (one included short-chained AHL molecules AB and the other long-chained AHL molecules DE) and four triple combinations as well as the quadruple mix of all tested AHL molecules. Plants were grown for additional 3 weeks and challenged with *P. syringae* pathovar *tomato* (*Pst*). Bacterial proliferation was assessed 96 h after the spray-inoculation. Since tri-partied systems are prone to variability, in addition to biological triplicates used in the experiment, the entire assay was performed independently and the results are presented in Figures 5A,B, respectively. Previous studies showed that pretreatment with single short-chained AHL had no impact on the resistance toward *Pst*, and pretreatment with a long-chained AHL induced an enhanced resistance to *Pst* via the AHL-priming (Schenk et al., 2012). Here, the proliferation of *Pst* revealed that our hypothesis was only partially correct. Indeed, pretreatment with all triple and the quadruple combinations induced enhanced resistance against *Pst*, if compared to the pretreatment with acetone (Figure 5). However, while comparing the bacterial proliferation on plants pretreated with the double AHL combinations and control, it was apparent that only the pretreatment with long-chained AHL (DE) induced enhanced resistance. The pretreatment with the short-chained AHL (AB) resulted in bacterial proliferation similar to control plants.

In summary, pretreatment with the complex triple or more AHL combinations induced enhanced resistance regardless of its composition, whereas a combination with only two short-chained AHL molecules was not able to induce AHL-priming for enhanced resistance.

## Discussion

The association between plant roots and bacteria possesses the potential to shape both, the host plant as well as the associated community. While the soil serves as the reservoir of a plethora of microbial species, it is the plant which models the microbial community in its rhizosphere and on the rhizoplane. Our knowledge regarding bacteria, which associate with plant increased significantly during the last decade, and lead to the definition of a core microbial community associated with plants, notably *Arabidopsis* (Bulgarelli et al., 2012; Lundberg et al., 2012). The diversity within the community as well as the influence of external factors (e.g., soil type) differs in different root-related compartments. Nevertheless, root exudates have the biggest influence on the structure of the rhizosphere community. Their composition was postulated as the driving force structuring microbial communities on many occasions (Zhalnina et al., 2018). The microbial community in turn may influence the physiology of the host plant. The presence of so-called plant growth promoting bacteria is an excellent example, another are soil-borne pathogens. In fact, diversified root microbiome may be one of the key players in plant health, preventing diseases (Berg et al., 2017; Durán et al., 2018).

In this study, we assessed how plants respond to the complex communication between bacteria, which takes place in such communities. As a model, we chose the well-known response of *Arabidopsis* to bacterial quorum sensing molecules from the *N*-acyl homoserine lactones (AHL) group. These molecules are usually used by Gram-negative bacteria in their intra- and inter-population communication and have well-documented impact on *Arabidopsis* and other plants (Hartmann et al., 2014; Schikora et al., 2016). Several reports have shown that AHL may promote root length or induce AHL-priming for enhanced resistance (Mathesius et al., 2003; Ortiz-Castro et al., 2008; Hartmann et al., 2014; Schikora et al., 2016; Zhao et al., 2020).

Here, we broadened our knowledge on the interactions between AHL molecules and the plant from linear interactions with single molecules to the outcome of complex interactions between multiple AHL molecules and the host plant. The multitude of AHL-producing bacteria that have been already identified on plant roots (Berg et al., 2002; Balasundararajan and Dananjeyan, 2019) implies that a host plant may indeed encounter more than a single AHL molecule, a situation which would necessitate a coordinated response.

One of the responses to the presence of AHL molecules was a modification in root morphology. The intermediate C10-HSL seemed to have the strongest effect on *Arabidopsis* and induced shortening of roots as well as increased formations of lateral roots and root hairs (Ortiz-Castro et al., 2008). Similarly, other AHL molecules like the short-chained C6-HSL and C8-HSL also increased root length (Von Rad et al., 2008; Schenk et al., 2012). Such modifications were observed also in other plants; for instance, in wheat and barley, where the presence of AHL increased root length and plant biomass (Rankl et al., 2016; Moshynets et al., 2019), or in mung bean, where AHL induced the formation of adventitious roots (Bai et al., 2012). The morphological response to multiple AHL molecules seems to be different from the response to a single AHL. Although we observed the previously reported enhanced root elongation after treatment with single short-chained AHL, their combinations (AD and BD) inhibited both, root length and plant biomass. On the contrary, the presence of all four tested AHL molecules didn’t change those parameters. Whether this outcome is a result of contrary physiological reactions, for example contra-balancing hormone levels, is still an open question. It is also probable that the downstream signaling cascade initiated by a particular AHL molecule might interfere with the signaling cascade initiated by another AHL molecule.

AHL-priming for induced resistance is another phenomenon, widely discussed as a consequence of AHL presence. It gained much attention recently as a possible target for breeding approaches and alternative plant protection strategy (Hernandez-Reyes et al., 2014; Shrestha et al., 2019; Wehner et al., 2019). AHL-producing bacteria and also AHL molecules were shown to induce AHL-priming and therefore protect plants from diseases (Bauer and Mathesius, 2004; Hartmann et al., 2014; Schikora et al., 2016). Very effective is the long-chained oxo-C14-HSL (Schenk et al., 2014), however, other AHL molecules also enhanced resistance against pathogens (Mathesius et al., 2003; Schuhegger et al., 2006; Liu et al., 2020). In addition to the response to single AHL molecules, our results suggest that the induction of AHL-priming could be the general response to multiple AHL molecules. Indeed, we observed here that the presence of all combinations, except the double short-chain AB combination, induces not only the expression of several defense-related genes but also the resistance against the foliar pathogens *P. syringae*. Whether the response is based on the presence of long-chained AHL molecules or whether the responses to for example oxo-C14-HSL, a known AHL-priming inducer, overwrites other responses requires further studies. It is also important to note that in recent report (Liu et al., 2020), AHL-induced enhanced resistance was observed after treatment with the short-chained AHL, oxo-C8-HSL. This indicates, that different experimental conditions may play a role. Resistance to pathogens is a crucial feature desired in crop plants and therefore, results obtained in this study could open new opportunities for modern strategies in plant protection. It is namely envisageable to use diverse AHL-producing strains as members of the applied inoculum.

Yet another discovery is very interesting in this context, the resistance to abiotic stresses (high salt concentrations) was enhanced after the treatment with oxo-C6-HSL (Barriuso et al., 2008; Zhao et al., 2020). Even though the exact mechanism of AHL-induced salt tolerance is not yet known, the possibility to modulate the plant tolerance to abiotic stresses like salt or drought using AHL is very interesting.

## Conclusion

It is important to note that the response of the model plant *A. thaliana* to the presence of different AHL molecules reflects only one level of the interactions in the rhizosphere. Nevertheless, our results indicate that the complex interactions of multiple AHL on plants may have surprisingly similar outcomes. Their combinations had a relatively low impact on the growth but seemed to induce resistance mechanisms. Our findings indicate that induced resistance against plant pathogens could be one of the major outcomes of AHL perception. Such findings are indeed very interesting since they open new possibilities for plant protection approaches and improve our knowledge on how complex bacterial communities may influence the host plant.

## Data Availability Statement

All datasets presented in this study are included in the article/Supplementary Material.

## Author Contributions

ASh and ASc designed the study and wrote the manuscript. ASh, MG, IO, and JK performed the experiments. ASh analyzed the data. All authors contributed to the article and approved the submitted version.

## Conflict of Interest

The authors declare that the research was conducted in the absence of any commercial or financial relationships that could be construed as a potential conflict of interest.

## Supplementary Material

FIGURE S1 Experimental approach. **(A)** The structures of *N*-acyl homoserine lactone (AHL) molecules used in this study. *N*-(3-oxohexanoyl)-*L*-homoserine lactone (oxo-C6-HSL) (A), *N*-(3-oxooctanoyl)-*L*-homoserine lactone (oxo-C8-HSL) (B), *N*-(3-oxododecanoyl)-*L*-homoserine lactone (oxo-C12-HSL) **(D)** and *N*-(3-oxotetradecanoyl)-*L*-homoserine lactone (oxo-C14-HSL) **(E)**. **(B)** Timelines of different experiments in this study. *Arabidopsis thaliana Col*-0 seeds were germinated on sterile 1/2-strength MS medium and grown for an additional week, before transplanting to fresh medium supplemented with single AHL or their combinations for: (i) additional 3 weeks for the measurement of growth parameters, or (ii) 3 weeks before the challenge with *Pseudomonas syringae* pathovar *tomato (Pst)*. Two-week old plants designated for expression analysis were transferred to liquid MS medium supplemented with single AHL or their combinations 3 days prior to the challenge with 100 nM flg22.

TABLE S1 Primers used in this study. List of primers used for quantitative PCR in this study.

TABLE S2 Measured values. Experimentally acquired values for root length, plant biomass and induction of gene expression. Data were used in Figures 1–4 as the basis for normalization. Values for acetone-pretreated plants were set to 100%.
