# Supplementary figures and images for: Impact of Quorum Sensing Molecules on Plant Growth and Immune System
Source: Front Microbiol. 2020 Jul 16;11:1545. doi: 10.3389/fmicb.2020.01545 (PMC7378388; doi:10.3389/fmicb.2020.01545)

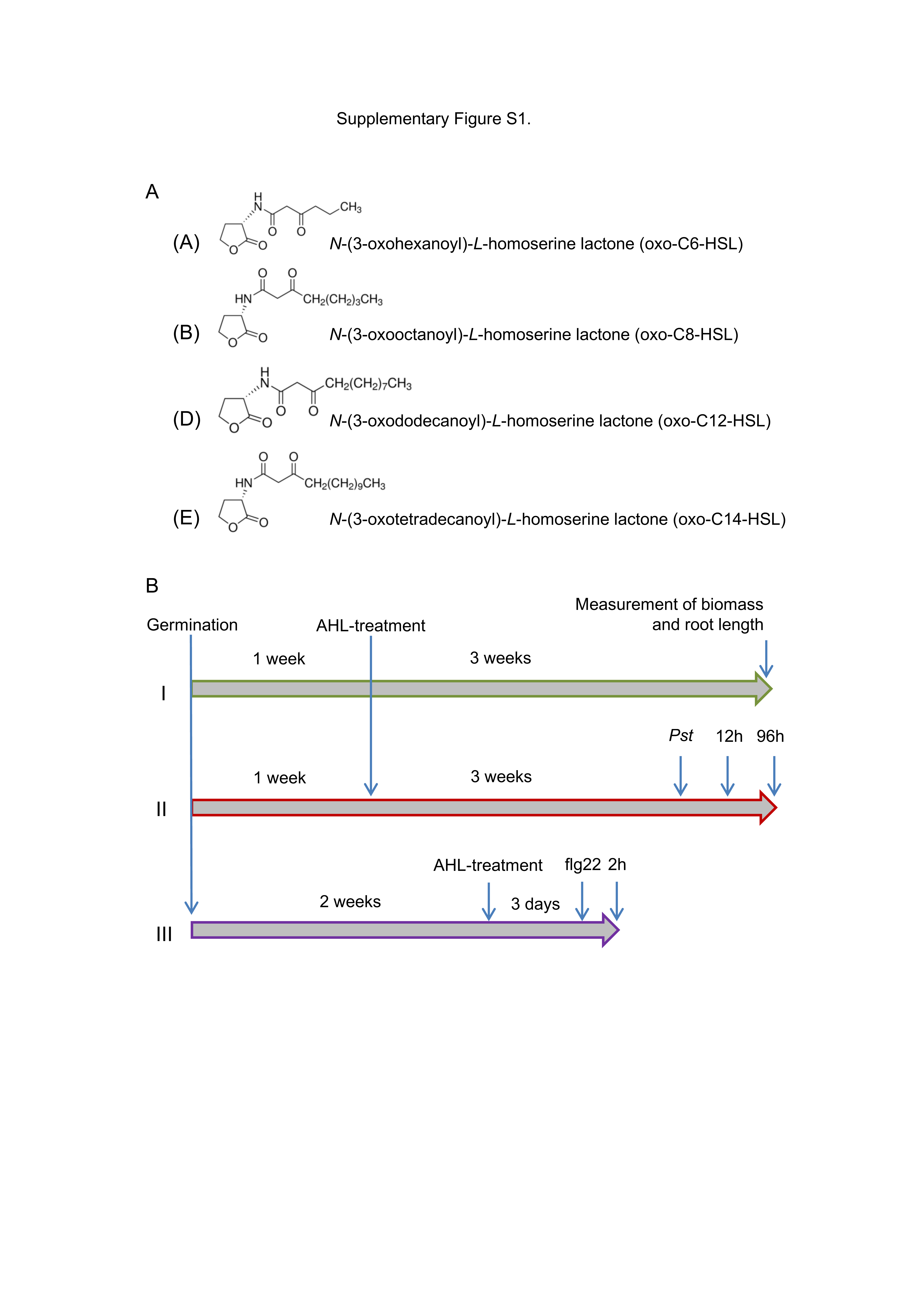

Supplement: FIGURE S1 — Experimental approach. (A) The structures of N-acyl homoserine lactone (AHL) molecules used in this study. N-(3-oxohexanoyl)-L-homoserine lactone (oxo-C6-HSL) (A), N-(3-oxooctanoyl)-L-homoserine lactone (oxo-C8-HSL) (B), N-(3-oxododecanoyl)-L-homoserine lactone (oxo-C12-HSL) (D) and N-(3-oxotetradecanoyl)-L-homoserine lactone (oxo-C14-HSL) (E). (B) Timelines of different experiments in this study. Arabidopsis thaliana Col-0 seeds were germinated on sterile 1/2-strength MS medium and grown for an additional week, before transplanting to fresh medium supplemented with single AHL or their combinations for: (i) additional 3 weeks for the measurement of growth parameters, or (ii) 3 weeks before the challenge with Pseudomonas syringae pathovar tomato (Pst). Two-week old plants designated for expression analysis were transferred to liquid MS medium supplemented with single AHL or their combinations 3 days prior to the challenge with 100 nM flg22. [file Image_1.TIF]
