# Supplementary material for: Impact of Quorum Sensing Molecules on Plant Growth and Immune System
Source: Front Microbiol. 2020 Jul 16;11:1545. doi: 10.3389/fmicb.2020.01545 (PMC7378388; doi:10.3389/fmicb.2020.01545)
Supplement: TABLE S1 — Primers used in this study. List of primers used for quantitative PCR in this study. [file Table_1.DOCX]

**Supplementary Material**

Supplementary Table S1. Primers used in this study

| **Oligonucleotide** | **Sequence (5´–3´)** | **Gene ID** | **Reference** |
| --- | --- | --- | --- |
| *UBQ4* fwd | GCT TGG AGT CCT GCT TGG ACG | *At5g25760* | Schikora et al. 2011 |
| *UBQ4* rev | CGC AGT TAA GAG GAC TGT CCG GC |  |  |
| *WRKY22* fwd | ATC TCC GAC GAC CAC TAT TG | *At4g01250* | Schikora et al. 2011 |
| *WRKY22* rev | TCA TCG CTA ACC ACC GTA TC |  |  |
| *WRKY29* fwd | TCC GGT ACG TTT TCA CCT TC | *At4g23550* | Schikora et al. 2011 |
| *WRKY29* rev | AGA GAC CGA GCT TGT GAG GA |  |  |
| *GST6* fwd | GCA CCT TGG AGT CAG TAC CC | *At2g47730* | Schenk et al. 2014 |
| *GST6* rev | GAT GGT CGG AAG AGG CAT AA |  |  |
| *Hsp70* fwd | CGC CAA CGA TCA AGG CAA CC | *At3g12580* | Schenk et al. 2014 |
| *Hsp70* rev | GCT TCT CAC CTG GAC CGG AA |  |  |
